# Impact of Atmospheric CO_2_ on Thermochemical Heat Storage Capabilities of K_2_CO_3_

**DOI:** 10.1021/acs.energyfuels.2c02886

**Published:** 2022-11-11

**Authors:** Natalia Mazur, Henk Huinink, Hartmut Fischer, Olaf Adan

**Affiliations:** † Department of Applied Physics, Eindhoven University of Technology, Den Dolech 2, 5600 MBEindhoven, The Netherlands; ‡ Eindhoven Institute for Renewable Energy Systems, Eindhoven University of Technology, P.O. Box 513, 5600 MBEindhoven, The Netherlands; § TNO Materials Solutions, High Tech Campus 25, 5656 AEEindhoven, The Netherlands

## Abstract

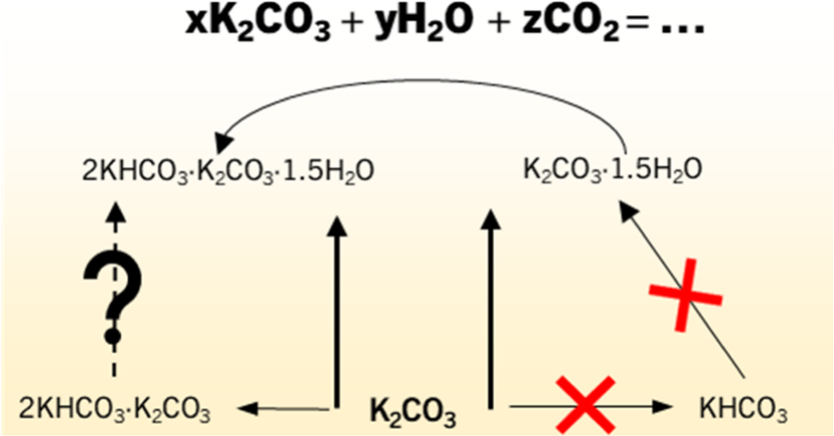

This work investigates the reactions occurring in K_2_CO_3_–H_2_O–CO_2_ under ambient CO_2_ pressures in temperature and vapor pressure ranges applicable for domestic thermochemical heat storage. The investigation shows that depending on reaction conditions, the primary product of a reaction is K_2_CO_3_·1.5H_2_O, K_2_CO_3_·2KHCO_3_·1.5H_2_O, or a mixture of both. The formation of K_2_CO_3_·1.5H_2_O is preferred far above the equilibrium conditions for the hydration reaction. On the other hand, the formation of double salt is preferred at conditions where hydration reaction is inhibited or impossible, as the thermogravimetric measurements identified a new phase transition line below the hydration equilibrium line. The combined X-ray diffraction, thermogravimetric analysis, and Fourier-transform infrared spectroscopy study indicates that this transition line corresponds to the formation of K_2_CO_3_·2KHCO_3_, which was not observed in any earlier study. In view of thermochemical heat storage, the formation of K_2_CO_3_·2KHCO_3_·(1.5H_2_O) increases the minimum charging temperature by approximately 40 °C. Nevertheless, the energy density and cyclability of the storage material can be preserved if the double salt is decomposed after each cycle.

## 1 Introduction

Potassium carbonate (K_2_CO_3_) is an abundant and non-toxic chemical with many applications.^1^ With the growing need for CO_2_ neutrality and sustainable energy solutions, K_2_CO_3_ has gained interest both as a thermochemical material (TCM) for domestic heat storage^2^ and as a CO_2_ capture material.^3^

When used for thermochemical heat storage (TCHS), the (de)hydration reaction, shown in reaction 1, is of interest.
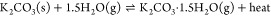
1

The hydration reaction of anhydrous K_2_CO_3_, also seen as the discharge reaction, is exothermic, and the released heat can be harvested for domestic heating or hot tap water. However, dehydration is endothermic. Therefore, heat needs to be supplied by, for example, solar thermal collectors to recharge the system. The water vapor transport to and from the salt is done with the aid of a carrier gas. It can be done with nitrogen, which is an inert gas, so it will not interact with K_2_CO_3_ or in a vacuum, where no other gases than water vapor are present during the reaction. Nevertheless, to use either of the solutions, a so-called closed system needs to be built,^4^ which does not allow for the exchange of gases with the environment. Heat is transported in and out of the system through a heat exchanger, which increases the system’s complexity and size as all reactants must be stored locally.

On the other hand, an open system can use humid, atmospheric air as the carrier gas. This system is less complex. However, using air as a carrier gas introduces other gases into the system, one of which is CO_2_.

The reaction between anhydrous or hydrated K_2_CO_3_ and CO_2_, often called the carbonation reaction, can result in the formation of, among others, potassium bicarbonate (KHCO_3_), according to reactions 2 and 3.
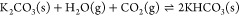
2
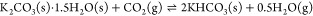
3

Both reactions have been extensively investigated for CO_2_ capture applications, where primarily reaction 2 is of interest. Most of the work has been done at temperatures of 40–60 °C and elevated CO_2_ pressures (*p*_CO_2__ ≥ 10 mbar), often with K_2_CO_3_ supported on an inert matrix,^5−14^ as the primary purpose of those investigations was to develop material for flue gas scrubbing.

Next, several works investigated CO_2_ capture with K_2_CO_3_-based materials at ambient or close to ambient conditions,^15−19^ which may reflect conditions encountered in a TCHS system. This extensive body of work resulted in a series of interesting observations.

First, the calcined KHCO_3_, which is KHCO_3_ heated up above 100 °C and converted to K_2_CO_3_ (reaction 2), is much more prone to re-carbonation than pristine K_2_CO_3_, and it is K_2_CO_3_ that has been prepared through recrystallization or dehydration of K_2_CO_3_·1.5 H_2_O.^7,8,12,15^ However, if pristine K_2_CO_3_ undergoes sufficient number of carbonation cycles (reaction 2), its sensitivity to CO_2_ increases.^13,14^

Second, the carbonation reaction is not the only possible reaction to occur. In addition, the formation of a double salt of potassium bicarbonate and sesquihydrate [K_4_H_2_(CO_3_)_3_·1.5H_2_O = 2KHCO_3_·K_2_CO_3_·1.5H_2_O] is also possible.^12^ The formation of double salt proceeds according to the following reactions
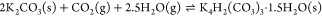
4
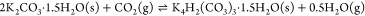
5

The precise reaction conditions determine whether KCHO_3_ or the double salt forms. In earlier studies, KHCO_3_ was usually observed at *p*_CO_2__ higher than the partial vapor pressure (*p*_vap_),^5,10,16^ whereas the double salt was more frequently observed at lower *p*_CO_2__.^17,18^ It is postulated that the formation of the double salt might be necessary for carbonation, according to reaction 6.^17,19^
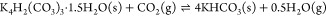
6

**Figure 1:**
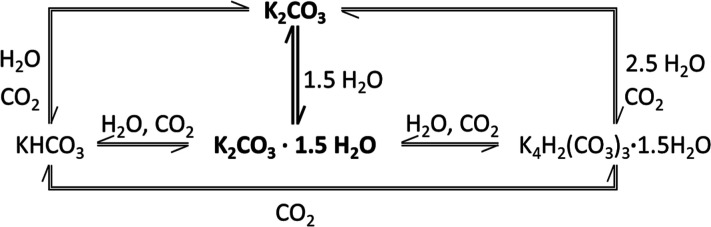
Equilibria in the K_2_CO_3_–CO_2_–H_2_O system with the hydration reaction highlighted in bold.

The reactions occurring in the K_2_CO_3_–H_2_O–CO_2_ system can be summarized in a series of equilibria presented in Figure 1. It shows that the utilization of K_2_CO_3_ as a CO_2_ capture material and a TCM are closely related. Both deal with the same reactants, and the energy involved in various equilibria is of interest. The reaction enthalpy determines the equilibrium conditions that outline the phases’ stability regions and the heat needed to regenerate the material.

An early study by Sögütoglu^20^ has considered the formation of KHCO_3_ as an unwanted side reaction with a negative impact on the performance and heat capacity of the TCHS system. A pressure–temperature region, where KHCO_3_ can form at ambient conditions, was calculated based on thermodynamic calculations. However, those did not consider the formation of the double salt. None of the recent studies on K_2_CO_3_ as a TCM has considered any possible side reactions at all.^21−24^ Because of the high potential of the salt hydrate as a TCM and the variety of system designs, we believe it is vital to gain insight into the processes that could occur during TCHS system operation.

This work studies the processes occurring when K_2_CO_3_ is exposed to humid air with ambient CO_2_ concentration (400 ppm, 0.4 mbar) at temperatures and vapor pressures applicable to the TCHS system. The goal is to determine which reactions are occurring under those conditions and to what degree they impact the TCM. The processes occurring in the system are studied by in situ X-ray diffraction (XRD) and Fourier transform infrared (FTIR) spectroscopy to determine the nature of phase transition and thermogravimetric and calorimetric methods to determine the impact of various phases on the transition temperatures and energy density.

## 2 Experimental Section

### 2.1 Sample Preparation

K_2_CO_3_ used in this study was obtained by calcining KHCO_3_ supplied by Evonik. The as-received powder was ground in a pestle and mortar, sieved to 50–164 μm fraction, and used as a precursor for anhydrous K_2_CO_3_ without further purification. The calcination was conducted either in an oven or in situ, and the completion of the process was based on the measured change in the sample mass.

### 2.2 Powder XRD

The development of new phases, when calcined KHCO_3_ was exposed to moist air, was monitored by powder XRD. The measurements were done in situ in a Rigaku Miniflex 600 X-ray diffractometer (Cu Kα source; Be monochromator, λ = 1.54 Å, 40 kV, 15 mA, D/tex Ultra2 1D detector). The conditions were controlled with an Anton Paar BTS500 benchtop heating stage. The temperature of that heating stage was calibrated with an external thermocouple. In addition, an external in-house built humidifier was coupled to the device to ensure fixed humidity conditions inside it. The humidifier was calibrated by determining hydration onset points of LiCl at 40, 50, and 60 °C.^25^ All measurements were done using a Ni sample holder under an 800 mL/h constant airflow. The measurements were done between 10 and 55 2θ with 0.01° step size and 5°/min scan speed. The measured patterns were matched against entries from the Crystallography Open Database (COD) and the Inorganic Crystal Structure Database (ICSD) with the help of Rigaku PDXL2 software.

First, ground and sieved KHCO_3_ was calcined in an oven set to 160 °C. Second, the sample was aligned in the sample holder and placed in the heating stage at 130 °C for 30 min under dry airflow to remove any moisture absorbed during sample preparation. Third, the stage was cooled down to the desired temperature and equilibrated in dry airflow for 30 min.

**Figure 2:**
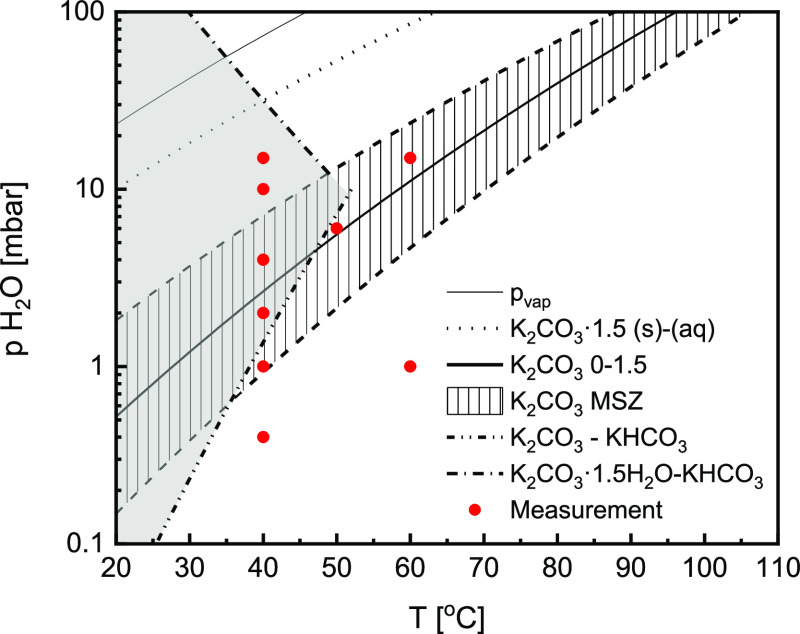
Phase diagram of K_2_CO_3_ adapted from refs (20) and (25), showing the measured K_2_CO_3_ hydration equilibrium (solid black line), the metastable zone where (de)hydration (reaction 1) is inhibited by a nucleation barrier (dashed area). The superimposed gray area shows the KHCO_3_ formation region based on calculated equilibrium lines for K_2_CO_3_·(1.5H_2_O)–KHCO_3_ transitions at 0.4 mbar CO_2_. Conditions for in situ XRD measurements are marked with red dots.

After the equilibration, the desired humidity was introduced into the chamber. Measurements were conducted at 40, 50, and 60 °C and at a fixed vapor pressure (*p*_vap_), as indicated by the red points in Figure 2. A diffraction pattern was collected every 30 min for the first four scans, then every 1 h for the subsequent two scans, and finally every 2 h for the final 20 h with a total measurement time of 24 h (excluding in situ drying).

### 2.3 Thermogravimetric Analysis

Thermogravimetric analysis (TGA) was done in a TGA 851e, Mettler-Toledo. The temperature of the device was calibrated using In, Zn, and benzophenone calibration standards by determining their melting temperature from the heat flow signal. An external humidifier was coupled to the TGA apparatus to control the humidity inside the TGA oven. The humidifier was calibrated by determining the deliquescence onset points at 25 °C of LiCl·H_2_O, MgCl_2_·6H_2_O, K_2_CO_3_·1.5H_2_O, and Mg(NO_3_)_2_·6H_2_O.^26^ All experiments were conducted at a fixed flow rate of 300 mL/h. Compressed air with a CO_2_ concentration of 350–400 ppm was used during the measurement.

Approximately 6 mg of KHCO_3_, resulting in approximately 4 mg of anhydrous K_2_CO_3,_ was used in experiments. For measurements, 40 μL aluminum pans from Mettler-Toledo were used. Each measurement started with in situ calcination at 190 °C.

The reaction onset points were determined with isobaric measurements by scanning between 130 and 20–50 °C at 1 K/min between 2 and 10 mbar. The measurements were later expanded by scanning the same temperature range with a cooling rate of 0.1 K/min at 5, 9, and 13 mbar. Finally, the reaction onset points were determined by determining the knee point in the first derivative of mass versus the measured sample temperature.

### 2.4 FTIR Spectroscopy

FTIR spectroscopy was used to probe several characteristic vibrational modes in carbonate materials. Measurements were conducted in an IRSpirit FTIR spectrophotometer with an attenuated total reflectance attachment from Shimadzu under ambient conditions. The FTIR measurements were conducted between 4000 and 650 cm^–1^. The presented data are an average of 10 consecutive scans.

Samples for FTIR were prepared in the TGA 851e to ensure the precise preparation conditions, following a similar procedure to earlier TGA measurements. First, approximately 6 mg of powder was calcined in dry air in situ for 30 min at 190 °C. Subsequently, the temperature was lowered to 40 °C and equilibrated in dry air for another 30 min. After that, the humid air with desired partial vapor pressure was introduced into the system and held constant for at least 2 h. Finally, the sample was moved to the FTIR spectrophotometer right after the measurement had ended.

### 2.5 Differential Scanning Calorimetry

The energy involved in the decomposition of byproducts was measured by differential scanning calorimetry (DSC) using the DSC822 apparatus from Mettler Toledo. The device was calibrated by determining the onset temperature and heat flow during melting benzophenone, In, and Zn standards.

Samples for DSC were prepared in an identical way as for FTIR. The sample was moved to the DSC right after the TGA measurement had ended. Measurements were done between −15 and 160 °C with a 1 K/min heating rate. The measurement was done under a dry N_2_ atmosphere with a fixed flow rate of 1.4 L/h.

## 3 Results

### 3.1 Reactivity at Ambient CO_2_ Conditions

In the first instance, the thesis brought forward by Sögütoglu et al.,^20^ who calculated a range of *p*–*T* conditions at which K_2_CO_3_ should convert to KHCO_3_ (-··-·· line in Figure 2), is tested. It is done through a series of isobaric measurements in air at selected vapor pressures by lowering the temperature at 1 and 0.1 K/min.

**Figure 3:**
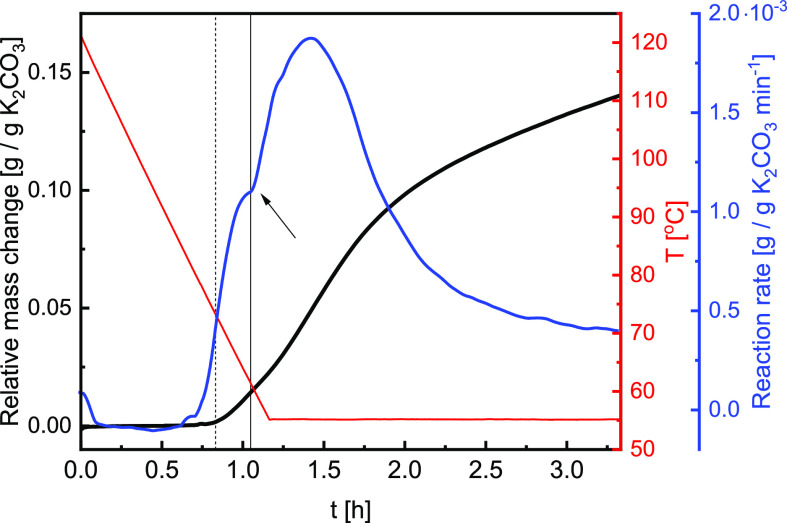
An example of an isobaric measurement at 12 mbar *p*_vap_ in air, showing the relative change in mass (black), the corresponding changes in the reaction rate (blue), and the measured sample temperature (red). The vertical lines mark the MSZ boundary for K_2_CO_3_·1.5H_2_O → K_2_CO_3_ transition (dashed) and equilibrium K_2_CO_3_ → K_2_CO_3_·1.5H_2_O transition (solid) at 12 mbar *p*_vap_. The arrow marks the second increase in the reaction rate.

Figure 3 presents data gathered at 12 mbar in air and 1 K/min scanning rate between 125 and 55 °C. Interestingly, the mass uptake (black plot) starts at conditions where no reaction was expected. Since this behavior does not correspond to any known or presumed transitions, it cannot be caused by either of reaction 1–3.

**Figure 4:**
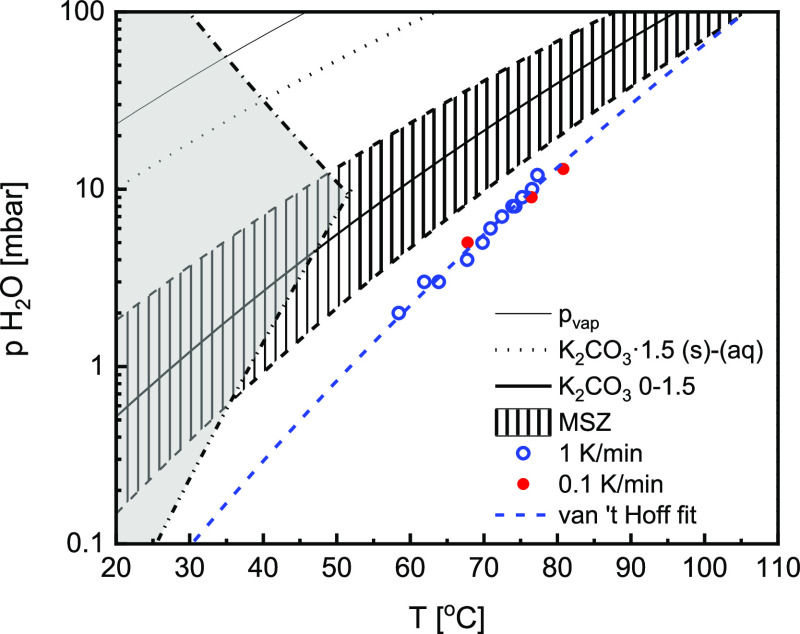
K_2_CO_3_ phase diagram with mass uptake points in air at fixed vapor pressures between 2 and 13 mbar marked with points measured at 1 K/min scanning rate (blue points) and 0.1 K/min scanning rate (red points). The blue dashed line is a van’t Hoff fit of the onset points.

This phenomenon was tested over a wider range of vapor pressures, as shown in Figure 4. The results follow the same trend in all cases as in Figure 3. The mass uptake starts at conditions below the dehydration metastable zone (MSZ) boundary (blue dots in Figure 4), and the measured reaction onset points form a new phase-transition line. The metastable zone is an area around hydration equilibrium conditions where the phase transition is hindered by a nucleation barrier and does not occur instantaneously despite thermodynamically favorable conditions.^25,27^ It means that reaction at conditions within MSZ will be preceded by an induction period, and only outside of that zone is the reaction instantaneous. In addition, we observe a second increase in the reaction rate (marked with an arrow in Figure 3), which aligns with K_2_CO_3_ (de)hydration equilibrium. This change in the reaction rate is also visible at other vapor pressures. It could be caused by the hydration of K_2_CO_3_ or a secondary process caused by CO_2_. Nevertheless, it is impossible to conclude which processes occur based on the observed mass change.

To test how sensitive the reaction onset point is to the driving force, similar measurements were repeated at three new vapor pressures (5, 9, and 13 mbar) and a lower cooling speed of 0.1 K/min (red dots in Figure 4). Since all measurements overlap well, it can be assumed that an instantaneous reaction occurs past those conditions since they indicate where mass uptake starts regardless of the scanning rate. Furthermore, it shows that it is possible to form a new compound under conditions previously regarded as a stable region for anhydrous K_2_CO_3._

The energy involved in this process can be estimated from the measured onset points by fitting them with a van’t Hoff plot. The fit resulted in Δ*H* = −87.6 kJ/mol and Δ*S* = −212.1 J/K mol, which are comparable with values calculated for reactions 1–3 presented in Table 1.

**Table 1:** Calculated Reaction Enthalpies Δ*H*_rx_ and Entropies Δ*S*_rx_ Based on Ref (28) and Mass Changes Δ*m*_rx_ Corresponding to the Reactions

reaction	Δ*H*_rx_ [kJ/mol K_2_CO_3_]	Δ*H*_rx_ [kJ/g K_2_CO_3_]	Δ*S*_rx_ [J/K mol]	Δ*m*_rx_ [g/g K_2_CO_3_]
(1) K_2_CO_3_ ⇌ K_2_CO_3_·1.5H_2_O	–96	–0.69	–216	0.196
(2) K_2_CO_3_ ⇌ KHCO_3_	–141	–1.02	–306	0.449
(3) K2CO3·1.5H_2_O ⇌ KHCO_3_	–44.6	–0.27^a^	–91	0.212^a^
(4) K_2_CO_3_ ⇌ K_4_H_2_(CO_3_)_3_·1.5H_2_O				0.323
(5) K_2_CO_3_·1.5H_2_O⇌K_4_H_2_(CO_3_)_3_·1.5H_2_O				0.106^a^
(6) K_4_H_2_(CO_3_)_3_·1.5H_2_O ⇌ KHCO_3_				0.096^b^

a Values calculated per g K_2_CO_3_·1.5H_2_O.

b Value calculated per g K_4_H_2_(CO_3_)_3_·1.5H_2_O.

### 3.2 Changes in Phase Composition with Varying Vapor Pressure

#### 3.2.1 XRD Study

**Figure 5:**
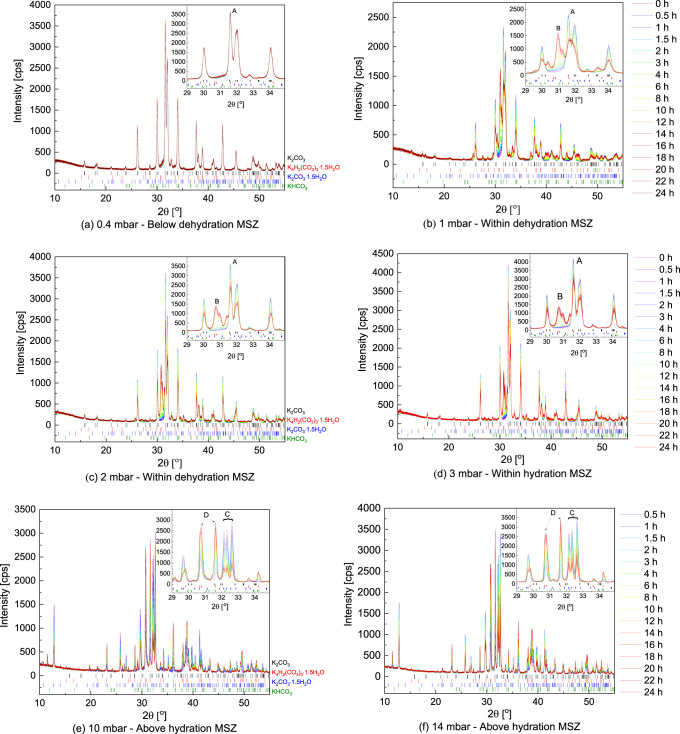
XRD patterns measured in air at 40 °C and fixed *p*_vap_ of (a) 0.4, (b) 1, (c) 2, (d) 3, (e) 10 and (f) 14 mbar. Scan colors change from purple to red with progressing measurement time. The letters mark characteristic peaks of A—K_2_CO_3_, B—unknown phase, C—K_2_CO_3_·1.5H_2_O, and D—K_4_H_2_(CO_3_)_3_·1.5H_2_O.

To better understand what kind of phases form at ambient conditions, a series of isobaric and isothermal XRD measurements were conducted in a range of vapor pressures and temperatures, as shown in Figure 2. In this section, measurements conducted at 40 °C and five different vapor pressures that correspond to four different regions in the phase diagram in Figure 2 are discussed: (1) below dehydration MSZ boundary (0.4 mbar), (2) below hydration equilibrium (1 and 2 mbar), (3) within hydration MSZ (3 mbar), and (4) above hydration MSZ (10 and 14 mbar). The data gathered at 40 °C are presented in Figure 5, while additional data collected at other temperatures can be found in the Supporting Information.

The initial phase analysis of calcined KHCO_3_ matches the single phase of anhydrous K_2_CO_3_ (COD 9009644).^29^ However, no peaks corresponding to KHCO_3_ or K_2_CO_3_·1.5H_2_O patterns were detected, so we conclude that KHCO_3_ has decomposed and did not react before the start of the measurement resulting in a well-defined starting material for our investigation.

**Figure 6:**
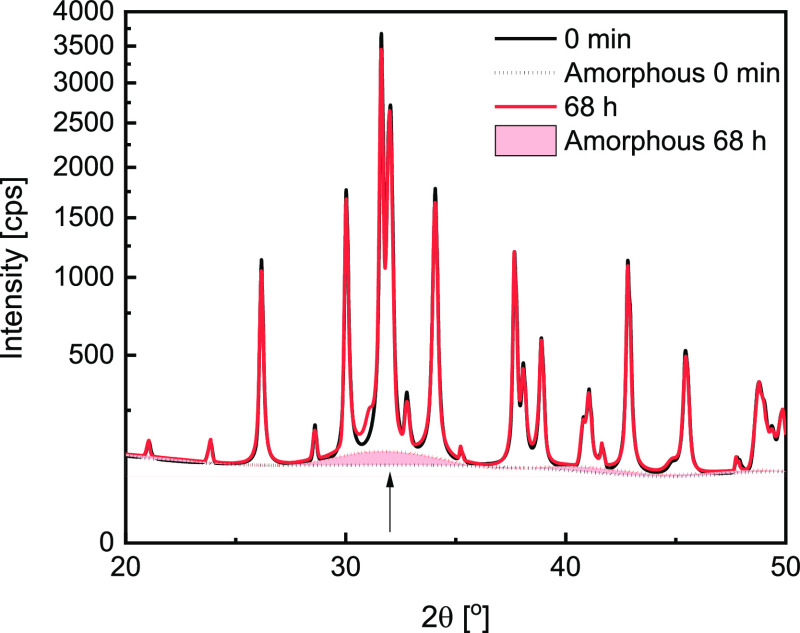
XRD patterns of K_2_CO_3_ measured at 40 °C and 0.4 mbar at the start of the measurement (black) and after 68 h of exposure to moist air (red) together with the calculated amorphous phase content (dotted lines). The black arrow points to the amorphous phase highlighted in red.

Starting at 0.4 mbar, which lies well above the presumed transition line established in Figure 4, no significant phase transformation was observed, even though the measurement was extended from 24 to 68 h. A closer investigation of the diffraction patterns in Figure 6, where intensity is plotted on a square-root axis, shows that with such low vapor pressures, the reaction proceeds through the formation of an amorphous phase which, after 68 h makes up 5% of the sample. It explains why there is no apparent change in the XRD patterns, although TGA measurements suggest that the reaction should occur. The amorphous phase transition becomes less prominent with increasing vapor pressure, where we see new reflexes appearing.

At 1 mbar, a decrease in intensity of the primary reflections of K_2_CO_3_ (Peak A—31.5–32.5° 2θ) and an appearance of a broad peak at 31° 2θ (Peak B) in the first 8 h of measurement can be seen. An increase in the amorphous content, similar to what we have observed at 0.4 mbar, is also observed. Only from the 10th h, new peaks belonging to a new crystalline phase are detected. The most significant phase transition occurs in the following 4 h. After that, the reaction rate seemingly stagnates as the pattern shows no significant changes. Identification of the peaks in the final pattern of this series shows the presence of anhydrous K_2_CO_3_ in combination with another phase that is difficult to differentiate due to overlapping patterns.

Similar phase transitions are visible at 2 and 3 mbar. The main difference between the measurements is the reaction rate. At higher vapor pressures, the reaction occurs faster, suggesting that partial vapor pressure is of great importance as it seemingly promotes the reaction. A similar transformation has been observed at higher temperatures (see the Supporting Information) when measurements were done within the hydration MSZ.

In neither of the cases was pure KHCO_3_ detected. The collected patterns from the final measurement cannot be matched against K_4_H_2_(CO_3_)_3_·1.5H_2_O (ICSD 401721) with complete confidence either, despite the good agreement for the double peak between 30.5 and 31.2° 2θ (Peak B). The faster reaction kinetics at higher partial vapor pressures point toward double salt formation (reaction 4), which requires 2.5 times as much water as CO_2_ to occur. Nevertheless, it suggests that mass uptake observed in earlier TGA measurements presented in Figure 4 is caused by simultaneous water and CO_2_ uptake that does not follow KHCO_3_ formation (reaction 2).

Finally, at conditions above MSZ (10 and 14 mbar), where hydration is no longer hindered, the first phase that forms is K_2_CO_3_·1.5H_2_O (ICSD 280789)^30^ with three characteristic peaks between 32 and 33° 2θ (Peak C). The conversion from anhydrate to hydrate seems complete within the first 30 min, as no peaks belonging to anhydrate are visible after the first measurement. Next to the prominent peaks belonging to K_2_CO_3_·1.5H_2_O, the formation of another phase is also detectable. In the case of the measurement conducted at 10 mbar, initial measurements show a double peak at about 31° 2θ, which then, with time, transitions toward a single peak at 31.5° 2θ (Peak D). The initial reflections that do not match K_2_CO_3_·1.5H_2_O are much like the pattern measured at lower humidities. During the measurement conducted at 14 mbar, only a single peak at 31.5° 2θ was observed from the beginning. Both phenomena are connected to the formation of the double salt. It shows that the growth of some of the crystal faces strongly depends on the environment in which the reaction is taking place. Next to the formation of the double salt, a decrease in the intensity of hydrate peaks is detected. As it happens at both 10 and 14 mbar, it shows that even at conditions when hydration is no longer inhibited, K_2_CO_3_·1.5H_2_O is not stable. Although it forms quickly at the start of the measurement, making up a significant portion of the sample, it is consumed with time.

This series of isobaric measurements confirmed that no direct carbonation also occurs at low vapor pressures and ambient CO_2_ conditions. Instead, an amorphous phase initially forms, transforming into a new crystalline phase with time. As the vapor pressure increases and hydration is no longer inhibited, the primary phase transition is the hydration reaction of K_2_CO_3_. Next to that, we observe the formation of the double salt. The content of that salt increases with time as K_2_CO_3_·1.5H_2_O is slowly consumed according to reaction 5. It shows that the equilibria presented in Figure 1 can occur in parallel. Given the structural complexity of the double salt, it is most likely that it is more kinetically hindered. Therefore, the reaction seems to be in competition with one another, and the precise reaction progress depends on the reaction conditions.

#### 3.2.2 TGA and FTIR Studies

**Figure 7:**
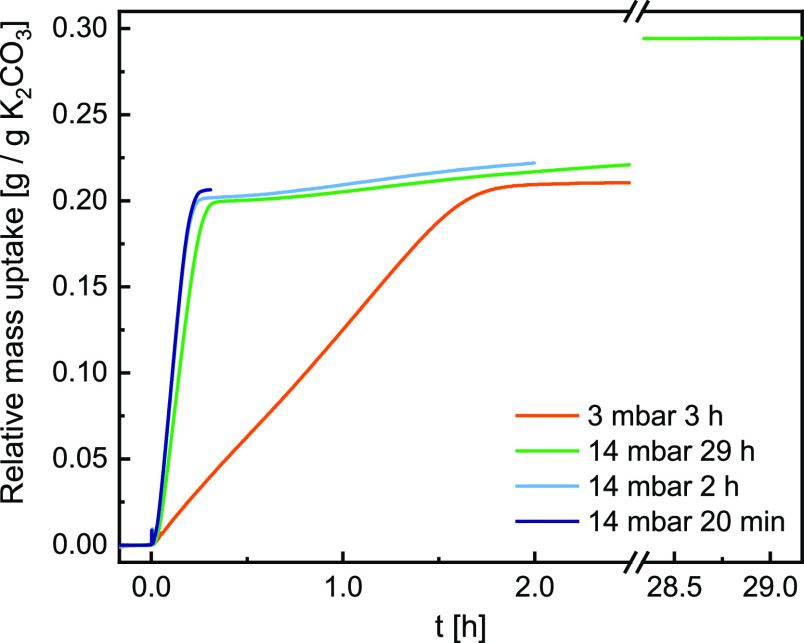
Relative mass uptake recorded in TGA in air at 40 °C and 3 and 14 mbar *p*_vap_ for a varying period of time indicated in the legend. Identical measurements were conducted for FTIR and DSC studies.

**Figure 8:**
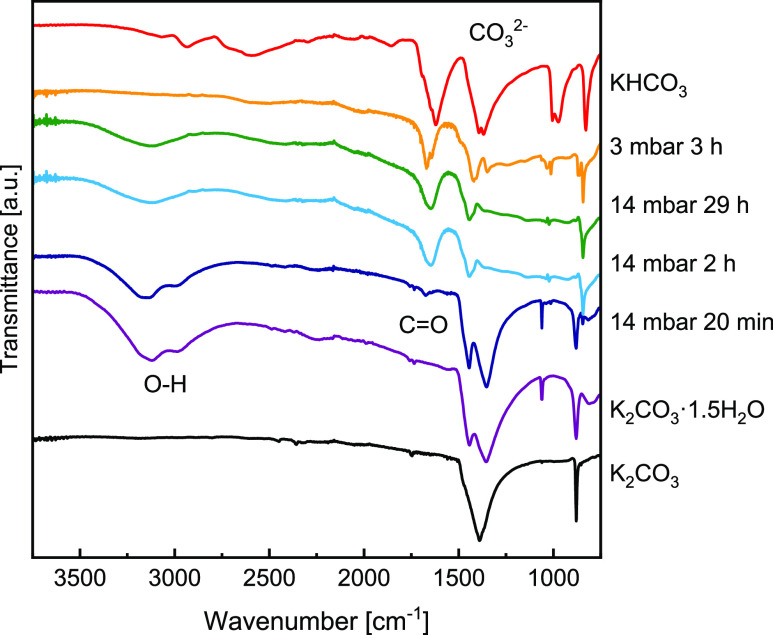
FTIR spectra recorded at ambient conditions on samples prepared in TGA at 3 and 14 mbar and pure reference compounds. Characteristic bonds under investigation are labeled in the figure.

Since the XRD measurements were inconclusive at low vapor pressures, and the final phase composition was difficult to define, TGA and FTIR spectroscopy were employed to gather more details about the reaction progress and its product. For this purpose, samples were prepared in TGA at well-defined conditions (40 °C and 3 and 14 mbar), and the obtained mass uptake curves are presented in Figure 7. 3 mbar vapor pressure was chosen as it provides the fastest conversion at conditions where the formation of K_2_CO_3_·1.5H_2_O is impossible. 14 mbar vapor pressure was selected as it gives the fastest hydration and subsequent double salt formation from the conditions investigated in XRD. By varying the time, a sample spends in TGA under those conditions, we aim to get more insight into this multistep reaction as a function of reaction progress. Samples prepared in the TGA apparatus were investigated by FTIR, together with pure anhydrous K_2_CO_3_, K_2_CO_3_·1.5H_2_O, and pure KHCO_3_ used as the reference. The analysis will focus on three characteristic bands in the carbonate spectra in Figure 8. First, an O–H stretching characteristic of water molecules is present at high wavenumbers (3500–2500 cm^–1^, marked with O-H). Second, at approximately 1650 cm^–1^, a band characteristic for bicarbonate is observed,^20^ which can be assigned to the C=O stretching.^31^ Third, there is the characteristic carbonate band at lower wavenumbers (1500–1200 cm^–1^, marked with CO_3_^2–^) which is due to the symmetric stretching of CO_3_^2–^.^32^ The difference in CO_3_^2–^ and HCO_3_ vibrational modes is caused by the lower symmetry of the bicarbonate ion.^33^

The changes in mass presented in Figure 7 show that the higher the humidity, the larger and faster the mass uptake. It adds to the XRD measurements, which have also shown faster reaction rates with increasing humidity. The ratio of water vapor to CO_2_ affects the equilibria presented in Figure 1, meaning that the individual reaction rates will be affected. Those equilibrium shifts and the materials they lead to are responsible for the changes in the curve slopes with progressing conversion. Next to reaction rate limitations, the phase transition can also be limited by nucleation rate or mass transfer previously observed for similar systems.^14^

Exposure to air with 3 mbar vapor pressure leads to a mass uptake of 0.2 g/g K_2_CO_3_. The XRD data in Figure 5d indicate that this is not due to hydration despite the mass change that could be expected from reaction 1. Moreover, the FTIR spectrum in Figure 8 (orange plot) does not show characteristic O–H stretching at high frequencies, meaning no water molecules are present in the structure. Another possibility is a two-step double-salt formation according to the following reactions
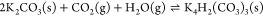
7
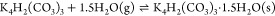
8

This hypothesis seems plausible as the mass uptake recorded after the TGA measurement only agrees well with the expected mass uptake for reaction 7. Moreover, a prominent C=O band at 1650 cm^–1^ is visible in the spectrum, confirming that bonds characteristic of bicarbonate are present in the material.

Two reactions were observed when the sample was exposed to 14 mbar vapor pressure, similar to XRD measurements. Initially, there was a fast mass uptake, adding up to 0.2 g/g K_2_CO_3_. The XRD data in Figure 5f show that K_2_CO_3_·1.5H_2_O forms as a primary phase, and the double salt is only a minor phase. As time progresses, K_2_CO_3_·1.5H_2_O transforms into the double salt observed in XRD and as a secondary, slow mass uptake in TGA. If we examine the second step closer, we see a dormant period of approximately 10 min after the initial mass uptake before the second step speeds up. Such a dormant period could be interpreted as an induction period and indicate a nucleation barrier for the second reaction. After 29 h, the recorded mass change is only 2% lower than the expected mass change during reaction 4. Thus, we conclude that the conversion of K_2_CO_3_ to double salt was nearly complete in the allocated time. The corresponding FTIR spectrum (green in Figure 8) shows O–H stretching at high wavenumbers, bicarbonate and carbonate bands, confirming that both H_2_O and CO_2_ are incorporated into the material.

A similar spectrum was recorded after 2 h exposure at 40 °C and 14 mbar, where the second reaction step was observed. However, only 20 min exposure to identical conditions results in a spectrum similar to K_2_CO_3_·1.5H_2_O and only a minor C=O band. It agrees well with the XRD data and confirms that at high humidities, reaction 1 is dominant at the start and is then followed by reaction 5. Whether or not a direct reaction of K_2_CO_3_ to double salt occurs (reaction 3) is difficult to conclude due to the speed of the other two reactions.

### 3.3 Impact of CO_2_ on Heat Storage Capabilities

In the previous sections, we have established that a combination of reactions can occur depending on reaction conditions and time. This section investigates the potential impact of CO_2_ on the performance of K_2_CO_3_ as a TCM. The investigation is subdivided into two categories. First, the impact of side reactions on the energy density of K_2_CO_3_ was investigated by employing DSC. Second, the reversibility of the reactions during repetitive (de)hydration cycles was probed with TGA.

#### 3.3.1 Transformation of K_2_CO_3_·1.5H_2_O

This section examines the impact of the sesquihydrate–double salt transformation on the system’s energy density. In a large system, the conversion often proceeds through a reaction front.^34^ It means that the material close to the inlet of the reactor is exposed to humid air until the entire reactor volume is converted. It has two immediate consequences for the system. First, sesquihydrate–double salt transformation consumes water, effectively lowering the humidity applied to the unreacted material. As a result, it might lead to lower power output as the reaction conditions are changed. Second, the formation of the double salt might impact the system’s energy density and charging temperature since the reaction enthalpy and decomposition onset points are unknown.

**Figure 9:**
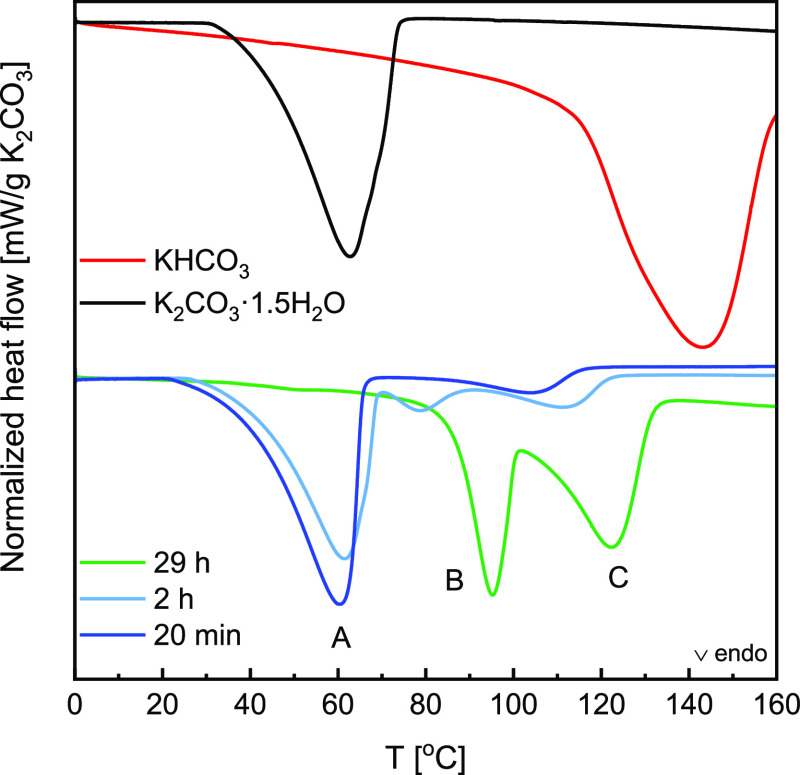
Normalized heat flow recorded in DSC during decomposition in dry N_2_ between 0 and 160 °C for samples prepared in TGA at 40 °C and 14 mbar for 20 min (dark blue), 2 h (light blue), and 29 h (green) together with pure reference compounds K_2_CO_3_·1.5H_2_O (black) and KHCO_3_ (red) measured under identical conditions. Corresponding mass uptake curves can be found in Figure 7.

Samples were prepared in TGA at well-defined conditions (40 °C, 14 mbar), and the final mass changes are tabulated in Table 2. For reference, the decomposition profiles of pure K_2_CO_3_·1.5H_2_O and KHCO_3_ recorded under identical conditions (0–160 °C, 1 K/min, dry N_2_) are also included. The thermograms presented in Figure 9 were collected from samples exposed to humid air (14 mbar, 40 °C) for 20 min, 2 h, and 29 h, as those three exposure times correspond to three conversions (see Figure 7).

**Table 2:** Measured Mass Loss Δ*m* and Reaction Enthalpy during Decomposition of Samples Prepared by TGA at 40 °C and 14 mbar for Three Different Exposure Times and Pure KHCO_3_ and K_2_CO_3_·1.5H_2_O as References

reaction time	measured reaction enthalpy [kJ/g K_2_CO_3_]	Δ*m* [g/g K_2_CO_3_]
29 h	–0.71	0.30
2 h	–0.74	0.22
20 min	–0.70	0.21
KHCO_3_	–1.00	0.44
K_2_CO_3_·1.5H_2_O	–0.68	0.19

Starting with the shortest exposure time (20 min), the primary reaction products are K_2_CO_3_·1.5H_2_O with a minor content of double salt. Therefore, it is reflected in two decomposition peaks. The first peak (peak A in Figure 9) agrees with the dehydration peak recorded for pure K_2_CO_3_·1.5H_2_O. Therefore, it implies that the decomposition of another compound must cause the second peak.

After the 2 h exposure, three distinct decomposition peaks are visible. First, there is dehydration of sesquihydrate at low temperatures, followed by two additional decomposition peaks, B and C. It shows that prolonged exposure to air leads to the formation of a compound that decomposes in two distinct steps.

Ultimately, the 29 h exposure, which, based on the mass change, resulted in nearly complete conversion to K_2_CO_3_·2KHCO_3_·1.5H_2_O, shows decomposition in two steps at higher temperatures than previous decomposition reactions. A two-step decomposition of the double salt has been previously reported at even higher temperatures,^35^ together with a minor shoulder at around 50 °C, which is also present in our measurement. This study postulated that the decomposition of K_2_CO_3_·2KHCO_3_·1.5H_2_O goes through the formation of K_2_CO_3_·2KHCO_3_·0.5H_2_O as it matched the observed mass loss. However, this theory contradicts the earlier proposed reactions 7 and 8, as the mass uptake corresponding to the formation of K_2_CO_3_·2KHCO_3_·0.5H_2_O or O–H stretching characteristic of the H_2_O molecule in the FTIR measurements was not observed. Nevertheless, it is possible that the double salt can have three hydration states, anhydrous, hemihydrate, and sesquihydrate, and their stability is highly sensitive to CO_2_ and H_2_O partial pressures. Unfortunately, based on present data, it is impossible to conclude which pathway the reaction takes, and it warrants further investigation.

By correlating the spectra gathered with FTIR in Figure 8 with the DSC data, we see that peak C appears first, together with the band characteristic of HCO_3_^–^. Furthermore, this band is present only after prolonged exposure. Therefore, we propose that peak C can be accredited to reaction 7, while peak B can be assigned to reaction 8.

Finally, Table 2 compares the reaction enthalpies based on the total area of the endothermic peaks. Both pure substances used as references show reaction enthalpy close to the theoretical values presented in Table 1. Thus, it can be assumed that the enthalpy measured from the decomposition after 29 h exposure is also close to the actual value of the pure double salt. It results in a reaction enthalpy of 98.5 kJ/mol K_2_CO_3_. Based on the crystal density of the double salt,^35^ the volumetric energy density is 1.2 GJ/m^3^, which is nearly identical to the volumetric energy density of K_2_CO_3_·1.5H_2_O.^2^ It shows that the formation of the double salt does not significantly impact the material’s gravimetric or volumetric energy density.

Nevertheless, it is somewhat lower than the enthalpy measured after a 2 h reaction time, suggesting that the hydration reaction stores more energy than the formation of double salts or that KHCO_3_ amounts below the XRD detection limit have been formed and are contributing to the measured heat flow. Furthermore, judging by the reaction onsets in Figure 9, this energy is stored at higher temperatures. For example, in dry N_2_, K_2_CO_3_·1.5H_2_O dehydrates at 32 °C, while the double salt decomposes at approximately 70–85 °C.

#### 3.3.2 Impact of Reaction Conditions

**Figure 10:**
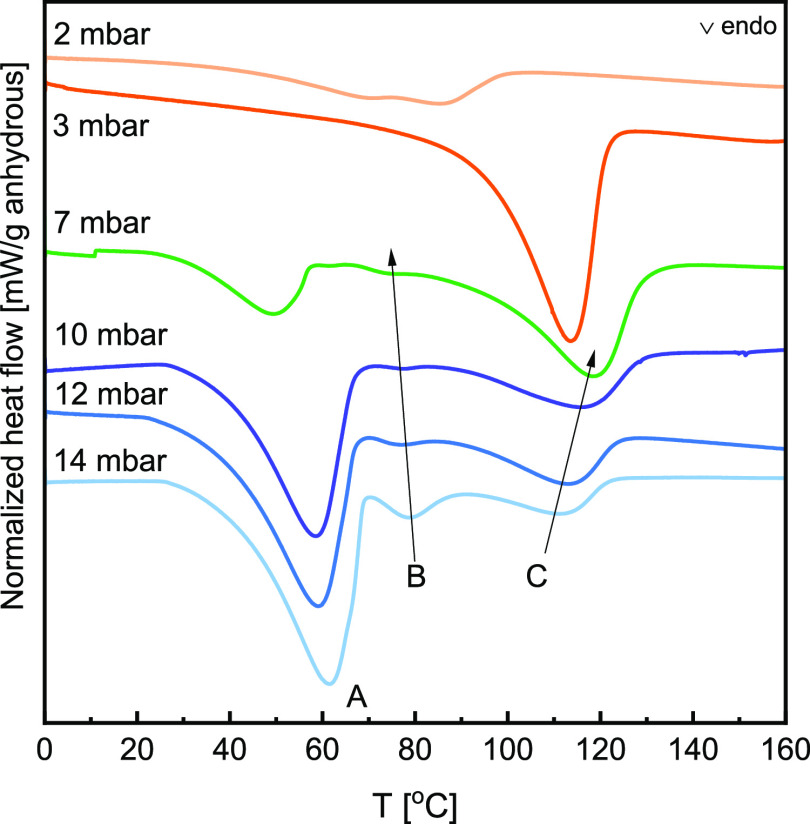
Normalized heat flow recorded in DSC during decomposition in dry N_2_ between 0 and 160 °C for samples prepared for 2 h by TGA at 40 °C and varying vapor pressures. The related mass changes are recorded in Table 3.

Based on earlier measurements, it can be presumed that below hydration MSZ (orange curves in Figure 10), K_2_CO_3_·2KHCO_3_ is the primary reaction product. The smallest heat flow signal, present as a double peak in the 38–101 °C range, was recorded from the sample exposed to 2 mbar vapor pressure. Since this sample showed the lowest mass uptake, it is not surprising that the heat released during decomposition is minor. However, it cannot be correlated with any of the reactions based on the decomposition temperatures. The large fraction of unreacted K_2_CO_3_ present in the material might affect the stability of the newly formed phases and decrease the decomposition temperatures. Moreover, the XRD study has shown that the reaction at low *p*_vap_ leads to the formation of an amorphous phase which might further impact the decomposition process.

When the sample was prepared within hydration MSZ at 3 mbar, only a single decomposition was observed with an onset at 93 °C. The decomposition temperature suggests that no K_2_CO_3_·1.5H_2_O or KHCO_3_ was formed, which decomposed at 31 and 113 °C, respectively. It also confirms the hypothesis that the formation of the double salt proceeds in two steps, where CO_2_ occlusion occurs first, given that the decomposition temperature is higher than the decomposition of K_2_CO_3_·2KHCO_3_·1.5H_2_O.

The double salt and sesquihydrate formation are expected at 7 mbar (green curve in Figure 10), the edge of MSZ. Finally, above hydration MSZ (blue curves in Figure 10), in the first instance, K_2_CO_3_ hydrates, followed by the transformation of the sesquihydrate into the double salt. By comparing the DSC curves with the preparation conditions, it can be seen that the size of the first endothermic peak (Peak A), which we have assigned to the dehydration reaction in the previous section, decreases with decreasing vapor pressure, and its onset shifts to lower temperatures.

In addition to the dehydration peak, two more peaks in the 70–138 °C region are recorded (labeled B and C in Figure 10), which are assigned to the two-step decomposition of the double salt. The intensity of peak B decreases, and its maximum shifts to lower temperatures with decreasing vapor pressure. Meanwhile, peak C increases in intensity and shifts to higher temperatures with decreasing vapor pressure. This dependence on the vapor pressure shows that the formation of the double salt, according to reactions 7 and 8, is favored at lower vapor pressures over the hydration reaction. However, there is a stronger competition between the hydration reaction and double salt formation at higher vapor pressures, either directly through reaction 4, reaction 5, or a combination of reactions 7 and 8.

Once again, the total energy released during decomposition was measured by DSC. Table 3 shows that the highest humidity results in the most significant mass uptake and the largest energy release, mainly originating from peaks A and B. When K_2_CO_3_ was exposed to lower vapor pressures, the water content in the final compound decreased, which can be inferred from decreasing peaks A and B. The variations in peak areas allow the estimation of the content of K_2_CO_3_·1.5H_2_O at the end of 2 h exposure. In the case of measurement at 14 mbar, sesquihydrate made up approximately 75% of the sample, and its content decreased to 70 and 66% at 12 and 10 mbar, respectively.

**Table 3:** Measured Mass Loss Δ*m* and Reaction Enthalpy during Decomposition of Samples Prepared by TGA for 2 h at 40 °C and a Series of Vapor Pressures

sample	measured reaction enthalpy [kJ/g K_2_CO_3_]	Δ*m* [g/g K_2_CO_3_]
2 mbar	–0.17	0.07
3 mbar	–0.49	0.21
7 mbar	–0.47	0.19
10 mbar	–0.59	0.20
12 mbar	–0.61	0.21
14 mbar	–0.65	0.22

#### 3.3.3 TGA Cycling in Air

**Figure 11:**
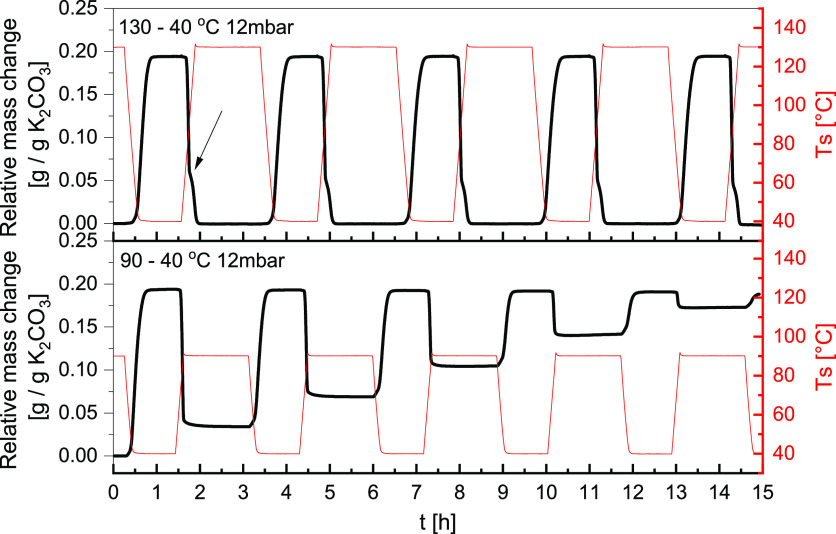
Isobaric cycling measurements at 12 mbar between top: 130–40 °C and bottom: 90–40 °C. Black plots show relative mass change, and red plots show the corresponding measured sample temperature. The black arrow shows the point where the decomposition slows down.

The impact of cycling conditions on phase formation and reaction reversibility will be investigated in this section. The measurements were conducted at isobaric conditions of 12 mbar. The temperature was scanned at 5 K/min between 40 and 130 °C (top Figure 11) or between 40 and 90 °C (bottom Figure 11). At the end of each temperature ramp, a 2 h temperature dwell was introduced to ensure complete conversion.

The evaluation starts with the cyclic behavior between 40 and 130 °C. In the top Figure 11, the reactions are fully reversible under those conditions. The mass uptake starts at approximately 55 °C and achieves a stable conversion within 20 min of reaching the temperature plateau. Since the mass is perfectly stable during the temperature dwell, we presume the extent of side reactions must be limited. The reverse reaction progresses in two steps. The first decomposition starts at 75 °C and accounts for 70% of the mass loss. At 90 °C (marked with an arrow on top in Figure 11), there is a drop in the decomposition rate. Then, the rate increases again at 114 °C, leading to the complete decomposition of the sample. This behavior is fully reproducible over five cycles. Because the mass registered during the 130 °C dwell is identical to the initial mass, we assume that all H_2_O and CO_2_ are removed from the sample, which reverts to anhydrous K_2_CO_3_. Since the decomposition progresses in two steps, it indicates that either two compounds are present in the sample after sorption at 40 °C and 12 mbar, or there is a single compound which decomposes in two discrete stages.

Since 130 °C might be a relatively high temperature to generate for some solar thermal collectors, the impact of lower decomposition temperatures is investigated. In the measurement presented at the bottom of Figure 11, the maximum decomposition temperature was set to 90 °C, which coincides with the previously observed decrease in the decomposition rate. The first mass uptake in this measurement is identical to the first cycle presented at the top of Figure 11. However, because the maximum decomposition temperature is set to 90 °C, not all products are decomposed. At the end of the first cycle, only 85% decomposition is achieved. Comparing it with the 25% double salt content estimated with DSC, we can assume complete dehydration and partial decomposition of the double salt. The decomposition degree decreases by approximately 20% with each cycle, resulting in only 10% decomposition by the fifth cycle. At this stage, the material can be considered nearly inactive.

Because less and less material decomposes with each cycle, it can be assumed that the double salt formed during previous cycles acts as a promotor for enhanced CO_2_ uptake for the subsequent cycles, thus amplifying the effect with each cycle. This measurement shows that the decomposition temperature strongly influences the cyclability of the material in air.

## 4 Discussion

During this research, a series of reactions of K_2_CO_3_ in the presence of water vapor and atmospheric CO_2_ were probed. A series of isobaric measurements in TGA have established that a reaction between K_2_CO_3_ and moist air starts at conditions where neither hydration nor carbonation reaction was expected. A new line within the phase diagram has been determined, which corresponded to a previously unexpected reaction onset. Based on the XRD measurements, the new line denotes an MSZ boundary for the new compound and not an equilibrium line, as the reaction has been observed at conditions before that line (60 °C, 1 mbar, see the Supporting Information). A further FTIR study of the phase transitions at low humidity has shown that this mass uptake is linked to the formation of C=O bonds characteristic of HCO_3_^–^. The XRD and TGA studies point toward the formation of a new compound, K_2_CO_3_·2KHCO_3_. The DSC measurement established that this salt decomposes in a single step at 93 °C. Nevertheless, it is highly hypothetical as it has not been observed in the literature before, and stoichiometry is inferred solely from TGA data. Due to the lack of conclusive XRD data, it cannot be confirmed at this point.

**Figure 12:**
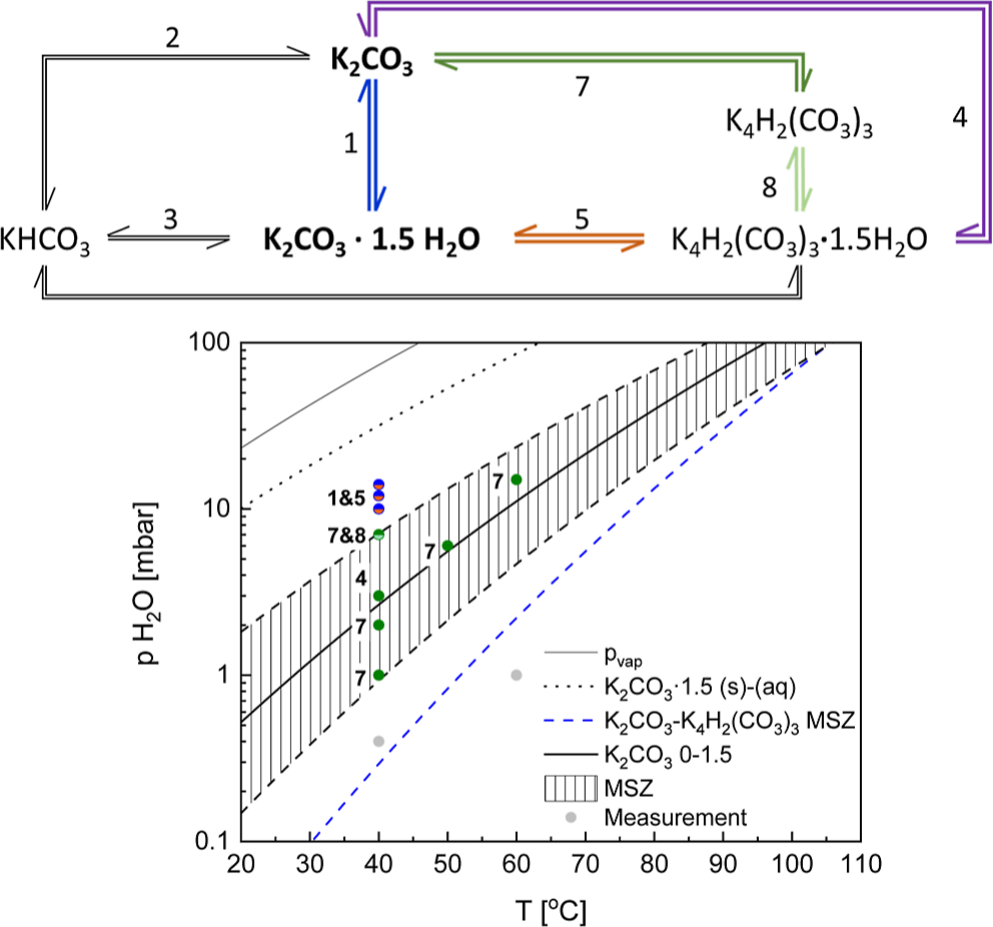
Top: Summary of reactions in the K_2_CO_3_–CO_2_–H_2_O system with reactions observed at ambient conditions highlighted in color. Bottom: Phase diagram showing TGA and XRD measurement conditions and the most probable reactions observed under those conditions color coded according to the figure above.

Figure 12 summarizes the observations from TGA and XRD measurements. At high humidities, that is, above the hydration MSZ, both K_2_CO_3_ hydration (reaction 1) and formation of a double salt K_2_CO_3_·2KHCO_3_·1.5H_2_O (reaction 4 or reactions 7 and 8) have been observed. The compound has been previously studied in the literature in its pure form.^35,36^ It has also been observed in many studies investigating K_2_CO_3_ for CO_2_ capture applications.^7,8,10,11,15−19,37,38^ Several of those works proposed that the formation of the double salt is an intermediate, perhaps even necessary, step for KHCO_3_ formation as it acts as an active species for further CO_2_ uptake.^10,19^ However, the current XRD study has not observed any subsequent KHCO_3_ formation. It is most likely due to the chosen reaction conditions, which, compared to earlier works, use a relatively low temperature, low *p*_CO_2_,_ and high *p*_vap_. Such conditions naturally shift the equilibrium toward double-salt formation (reaction 4 or 7) instead of carbonation (reaction 2). In the initial phase of the reaction, hydration (Figure 5e,f) and a minor double salt formation have been observed. The phase ratio changes with prolonged exposure to humid air, as the K_2_CO_3_·1.5H_2_O content decreases and the double-salt content increases (reaction 5). The speed at which the exchange happens depends on the partial vapor pressure. Those findings agree with other works that investigated K_2_CO_3_ as a CO_2_ capture material at ambient CO_2_ conditions.^39^

When all of K_2_CO_3_ is converted to double salt, the energy stored in the material is comparable to that stored in K_2_CO_3_·1.5H_2_O. However, if the conversion is only partial, as measured by DSC after 2 h of exposure in TGA, the heat released during decomposition is up to 13% lower, although the mass uptake is nearly identical. It is most likely linked to the multistep formation of the double salt through the abovementioned K_2_CO_3_·2KHCO_3_, which has an energy density that is 27% lower than sesquihydrate and 31% lower than the double salt. Furthermore, the complete transformation of K_2_CO_3_ anhydrous or hydrate to double salt is a very slow process. Communications which observed similar poor kinetics for the same reaction have accredited it to mass-transfer limitations.^14^ It means the partial formation of the double salt will most likely lower the energy density of the system and increase the charging temperature, as its decomposition starts between 70 and 85 °C, which is 40 °C higher than dehydration of pure K_2_CO_3_·1.5H_2_O in dry N_2_.

The need for elevated charging temperatures when working with K_2_CO_3_ in humid air became obvious during cyclic, isobaric measurements in air. At 12 mbar *p*_vap_, we observed a single mass uptake with the onset at approximately 50 °C. The decomposition proceeded in two steps, with the major mass loss at 76 °C and a subsequent, second and slower step at 105 °C. Five consecutive cycles were performed without any performance loss when the decomposition temperature was set to 130 °C. However, when the maximum decomposition temperature was lowered to 90 °C, only 85% decomposition was achieved after the first cycle, and within five cycles, nearly the entire K_2_CO_3_ was rendered inactive. This measurement has shown that once the double salt is formed, it acts as an inert material if not decomposed, and it also promotes further double salt formation since, with each cycle, the decomposition degree is lowered.

## 5 Conclusions

This work investigated the impact of atmospheric CO_2_ on K_2_CO_3_ as a TCM. During the investigation, the conditions at which K_2_CO_3_ can react with humid air, the products of those interactions, and their impact on the energy density were evaluated. The study shows that anhydrous K_2_CO_3_ can react with CO_2_ and H_2_O even at extremely low humidities (<1% RH), where an amorphous phase is formed. Based on the TGA, FTIR, and DSC data gathered at humidities below the hydration equilibrium, we propose a new form of a double salt, K_2_CO_3_·2KHCO_3,_ is formed. When the nucleation barrier does not inhibit the hydration, we observe the parallel formation of K_2_CO_3_·1.5H_2_O and K_2_CO_3_·2KHCO_3_·1.5H_2_O, followed by slow transformation of sesquihydrate into double salt.

From a TCHS application point of view, K_2_CO_3_ can be used in a reactor that uses air as a carrier gas. Such operation will not lead to any energy loss because the energy density of K_2_CO_3_·2KHCO_3_·1.5H_2_O is comparable with that of K_2_CO_3_·1.5H_2_O. Discharge should be conducted at as high vapor pressures as possible to minimize the effects of the secondary reactions to ensure fast conversion with the equilibrium shifted toward hydration. The charging should take place at temperatures above 100 °C to guarantee the double salt’s complete decomposition and maintain the system’s energy density.

Nevertheless, a more detailed analysis of the intermediate phases and their stability regions is needed to design the most optimal cycles for the heat storage system and avoid material degradation. Moreover, a more thorough crystallographic study should be conducted to elucidate the potential intermediate hydrates of the double salt.

## Supporting Information Available

In situ XRD spectra measured at 50 °C and 6 mbar, 60 °C and 15 mbar, and 60 °C and 1 mbar (PDF)
