# Supplementary material for: Impact of Atmospheric CO2 on Thermochemical Heat Storage Capabilities of K2CO3
Source: Energy Fuels. 2022 Nov 11;36(23):14464–75. doi: 10.1021/acs.energyfuels.2c02886 (PMC9720727; doi:10.1021/acs.energyfuels.2c02886)
Supplement: Supplementary file 1 — ef2c02886_si_001.pdf [file ef2c02886_si_001.pdf]

Supplementary information

Impact of atmospheric CO_2 on thermochemical heat storage capabilities of K_2CO_3.

Authors: *Natalia Mazur[1,2], Henk Huinink[1,2*], Hartmut Fischer[3], Olaf Adan[1-3]*

[1]Department of Applied Physics, Eindhoven University of Technology, Den Dolech 2, 5600 MB Eindhoven, The Netherlands.

[2]Eindhoven Institute for Renewable Energy Systems, Eindhoven University of Technology, PO Box 513, 5600 MB Eindhoven, The Netherlands

[3]TNO Materials Solutions, High Tech Campus 25, 5656 AE Eindhoven, The Netherlands

In-situ XRD spectra measured at 50 °C and 6 mbar, 60 °C and 15 mbar and 60 °C and 1 mbar and the ideal XRD patterns generated from CIF files for the pure phases.

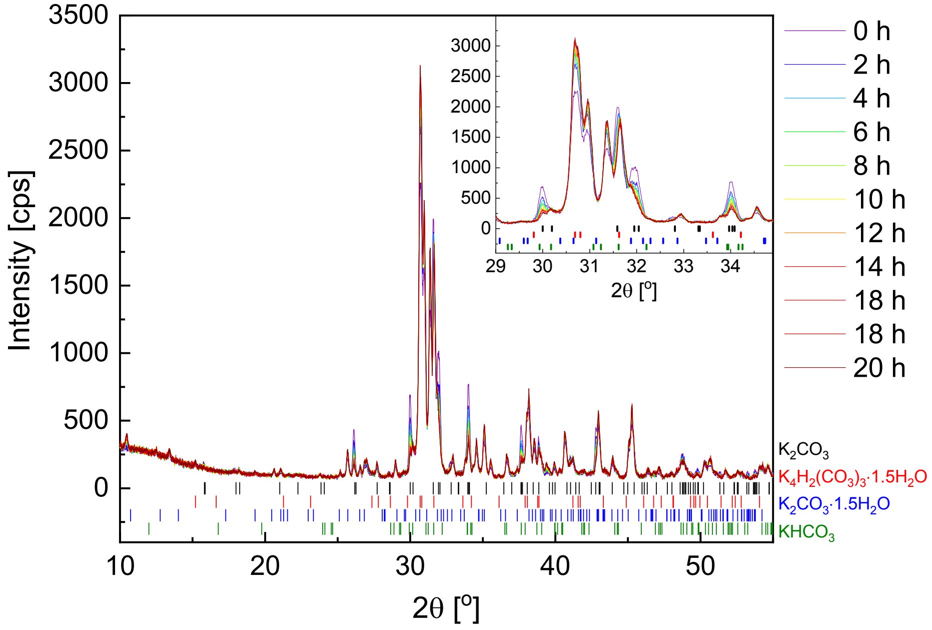

a) 50 °C 6 mbar - Hydration equilibrium

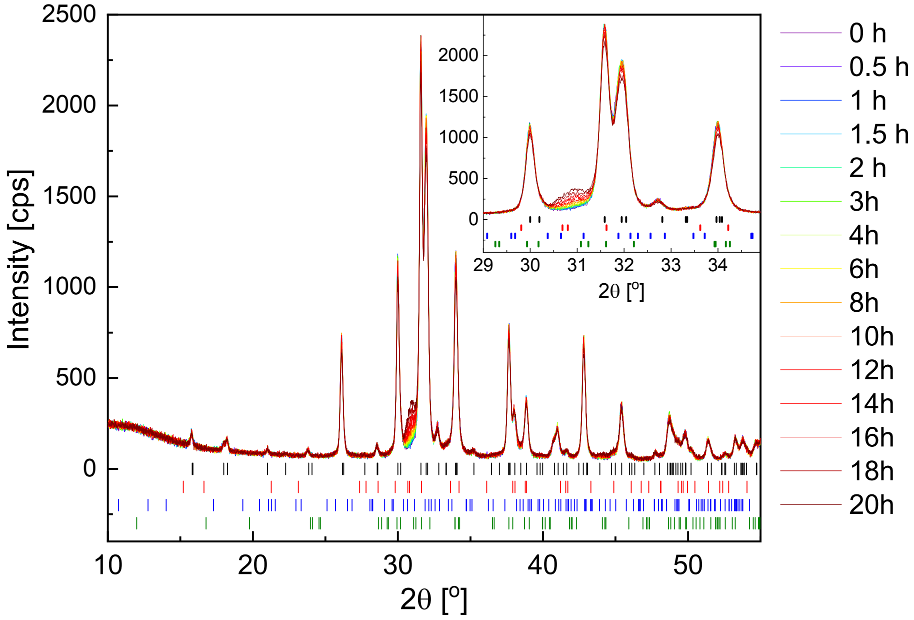

b) 60 °C 1 mbar - Below double salt MSZ

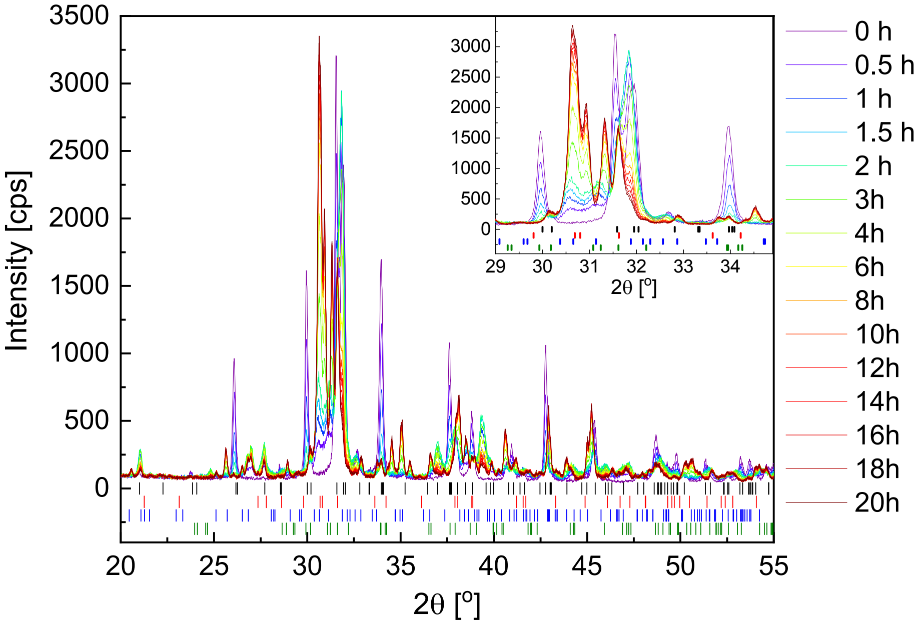

c) 60 °C 15 mbar - Within hydration MSZ

Figure S1. XRD patterns measured in air a) 45 °C 6 mbar, b) 60 °C 1 mbar c) 60 °C 15 mbar. Scan colours change from purple to red with progressing measurement time.

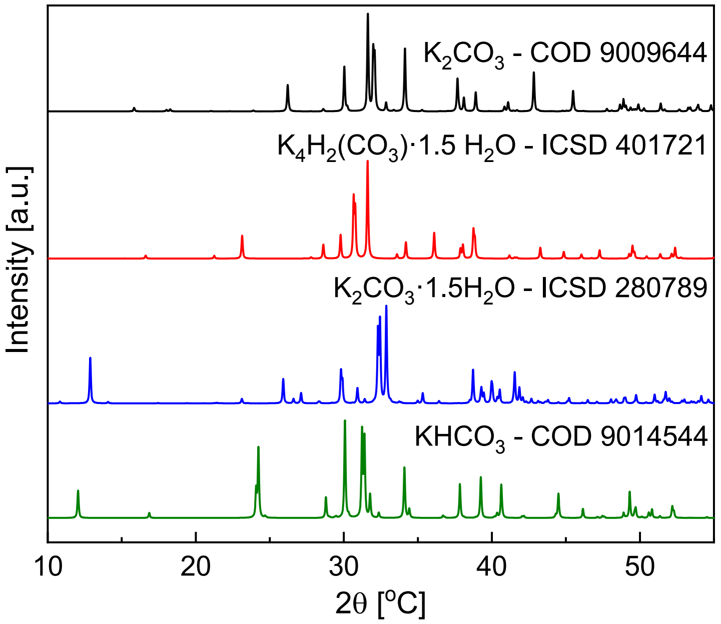

Figure S2. Ideal XRD patterns generated from corresponding CIF files.
